# Supplementary material for: Recurrent loss of CenH3 is associated with independent transitions to holocentricity in insects
Source: eLife. 2014 Sep 23;3:e03676. doi: 10.7554/eLife.03676 (PMC4359364; doi:10.7554/eLife.03676)
Supplement: Figure 1—source data 1. — DOI: http://dx.doi.org/10.7554/eLife.03676.004 [file elife03676s001.pdf]

Order	Species name	Source of genome/transcriptome/proteome	as current as/ version
DIPTERA	*Drosophila melanogaster*	http://flybase.org/	Dec-14
DIPTERA	*Aedes aegyptii*	http://flybase.org/	Dec-14
LEPIDOPTERA	*Bombyx mori*	http://silkworm.genomics.org.cn/silkdb/	SilkDB V2.0
LEPIDOPTERA	*Danaus plexippus*	http://reppertlab.org/monarchbase/	Assembly v3
LEPIDOPTERA	*Plutella xylostella*	http://dbm.dna.affrc.go.jp/px/	Feb-14
LEPIDOPTERA	*Manduca sexta*	https://www.hgsc.bcm.edu/arthropods/tobacco-hornworm-genome-project	Assembly v1.0
LEPIDOPTERA	*Heliconius melpomene*	http://www.butterflygenome.org/	Assembly v1.1
HYMENOPTERA	*Nasonia vitripennis*	http://hymenopteragenome.org/nasonia/	Nvit_1.0
HYMENOPTERA	*Bombus impatiens*	http://hymenopteragenome.org/beebase/	Bimp_2.0
HYMENOPTERA	*Apis mellifera*	http://hymenopteragenome.org/beebase/	Amel_4.5
HYMENOPTERA	*Atta cephalotes*	http://hymenopteragenome.org/ant_genomes/	Acep_1.0
COLEOPTERA	*Tribolium castaneum*	http://beetlebase.org/	13-Dec
HEMIPTERA	*Acyrtosiphon pisum*	http://www.aphidbase.com/	Version 2.1
HEMIPTERA	*Rhodnius prolixus*	https://www.vectorbase.org/organisms/rhodnius-prolixus	Feb-14
PHTHIRAPTERA	*Pediculus humanus corporis*	NCBI Genbank	JCVI_LOUSE_1.0

Figure 1- supplemental source data 1
