# Supplementary material for: Recurrent loss of CenH3 is associated with independent transitions to holocentricity in insects
Source: eLife. 2014 Sep 23;3:e03676. doi: 10.7554/eLife.03676 (PMC4359364; doi:10.7554/eLife.03676)
Supplement: Figure 1—source data 2. — Order, species name, centromere type (holocentromere [H] or monocentromere [M]), number of reads for the assembly, obtained transcripts longer than 250 base-pairs (only one transcript isoform) and number of significant tblastn alignments (E value 10–10) out of 16,644 annotated T. castaneum and 30,305 annotated D. melanogaster proteins. DOI: http://dx.doi.org/10.7554/eLife.03676.005 [file elife03676s002.pdf]

Order	Species	Centromere type	Reads (total)	Single end 100bp	Single end 150bp	Paired end 50bp	Paired end 100bp	454 reads	Transcripts (>250 bp)	Predicted number of proteins Query: *T. castaneum*	Predicted number of proteins Query: *D. melanogaster*
DIPTERA	*Drosophila melanogaster*	M	8769862	/	/	/	4384931	/	15040	4605	6309
COLEOPTERA	*Chrysochus auratus*	M	11061439	3284909	/	/	3888265	/	21467	6096	5025
COLEOPTERA	*Cyrtepistomus castaneus*	M	24784232	/	/	/	12392116	/	24956	7122	5946
COLEOPTERA	*Labidomera clivicollis*	M	6935044	6935044	/	/	/	/	16705	6079	5124
COLEOPTERA	*Megacyllene robiniae*	M	4245152	4245152	/	/	/	/	7992	4002	3388
COLEOPTERA	*Plagiodera versicolora*	M	1907103	1907103	/	/	/	/	9275	4133	3510
COLEOPTERA	*Rhyssomatus lineaticollis*	M	12012820	3417256	/	/	4297782	/	17593	6576	5456
COLEOPTERA	*Tetraopes tetraophthalmus*	M	8273718	8273718	/	/	/	/	15525	6261	5128
COLEOPTERA	*Anoplophora glabripennis*	M	126613384	/	/	/	63306692	/	23482	7867	6386
COLEOPTERA	*Leptinotarsa decemlineata*	M	87064144	/	/	/	43532072	/	35657	8026	6482
LEPIDOPTERA	*Cycnia tenera*	H	11258986	/	/	/	5629493	/	16643	5400	4180
LEPIDOPTERA	*Danaus erismus*	H	11756762	1308633	/	/	5878381	/	10709	5091	4138
LEPIDOPTERA	*Danaus gillipus*	H	3476486	/	/	/	1738243	/	9607	4568	3594
LEPIDOPTERA	*Euchaetes egle*	H	19883080	5905128	/	/	6988976	/	23427	7296	5622
LEPIDOPTERA	*Lophocampa caryae*	H	4958026	4958026	/	/	/	/	17455	5655	4500
LEPIDOPTERA	*Limenitis archippus*	H	3933071	3933071	/	/	/	/	9758	4383	3588
LEPIDOPTERA	*Lycorea halia atergatis*	H	18968673	16067453	/	/	1450610	/	11655	4249	3233
LEPIDOPTERA	*Papilio glaucus*	H	25863591	25863591	/	/	/	/	19000	7096	5690
LEPIDOPTERA	*Trichordestra legitima*	H	9474598	/	/	/	4737299	/	17576	6336	4955
HEMIPTERA	*Aphis nerii*	H	28196792	/	/	/	14098396	/	17381	6276	5924
HEMIPTERA	*Boisea trivittata*	H	4858096	4858096	/	/	/	/	13718	4793	4483
HEMIPTERA	*Lygaeus kalmii*	H	51924504	9476280	/	/	21224112	/	32157	5994	5650
HEMIPTERA	*Oncopeltus fasciatus*	H	57681394	7888794	/	/	27109674	/	42184	6156	5734
DERMAPTERA	*Anisolabis maritima*	H	87562912	/	/	43781456	/	/	26353	6401	6004
DERMAPTERA	*Forficula auricularia*	H	119368478	37973906	80044415	/	/	1350152	75242	7682	7061
ORTHOPTERA	*Acheta domesticus*	M	82905718	/	/	41452859	/	/	27393	6271	5882
PHASMATODEA	*Sipyloidea sipylus*	M	262246196	/	/	131123098	/	/	48280	6672	6063
BLATTODEA	*Blatella germanica*	M	244693922	/	/	122346961	/	/	47592	6239	5806
ODONATA	*Ladona fulva*	H	77135056	/	/	/	38567528	/	27320	6283	5871
ODONATA	*Libellula vibrans*	H	81875594	/	/	40937797	/	/	49642	7074	6778
EPHEMEROPTERA	*Ephemera danica*	M	105860062	/	/	/	52930031	/	48321	6601	6174

Figure 1- supplemental source data 2
