# Supplementary material for: Recurrent loss of CenH3 is associated with independent transitions to holocentricity in insects
Source: eLife. 2014 Sep 23;3:e03676. doi: 10.7554/eLife.03676 (PMC4359364; doi:10.7554/eLife.03676)
Supplement: Figure 2—source data 1. — DOI: http://dx.doi.org/10.7554/eLife.03676.016 [file elife03676s003.docx]

| **GENE** | **ORDER** | **SPECIES** | **ID** | **SEQUENCE** |
| --- | --- | --- | --- | --- |
| CenH3 | DIPTERA | *Drosophila melanogaster* | FBpp0086787 |  |
| CenH3 | DIPTERA | *Aedes aegyptii* | AAEL009296 |  |
| CenH3 | COLEOPTERA | *Tribolium castaneum* | TC012577 |  |
| CenH3 | COLEOPTERA | *Chrysochus auratus* | Locus_16594 | MPRRKSSPKKNKSPKKTTVAYRVSSKQFKVNVTTIADIKRLQNSTKLCIPKLPFSKLIRELLMKYTTINYRVEFRALEALQEAAEIYTTQLFEDSNRCAYHARRITVQPRDMQLALSIRGPSDPGYS |
| CenH3 | COLEOPTERA | *Cyrtepistomus castaneus* | Locus_22977 | MRTKNKSPGKVTKRKIMNYVVHNMQTSCPMSTYKEMRRLQNHTDLLIPKLPFSRLIREIMMEFGASHNMQGLALQALHEAAEQYLVQLLSDANRCCAHGNRVTVAPKDMRLVLEIRGVRDPG |
| CenH3 | COLEOPTERA | *Labidomera clivicollis* | Locus_1765 | MVNRKSTPKKKKAPSKQVTKIFKLHNKNYKINVTTLREIKRLQSTTSTLIPRLPFARIIREILMQYTTPDHRVQLEALRALQEAAELYLVQLFEDANRCAYHAKRVTLKPNDMNLVLEIRGTSDPGYS |
| CenH3 | COLEOPTERA | *Megacyllene robiniae* | unassembled reads | KVAVTTLREIRKLQSTTKLCIPRLPFSRLIREIEIYLTYLFEDANRCAHHAKRVTVKPNDMRLVLEIKG |
| CenH3 | COLEOPTERA | *Plagiodera versicolora* | Locus_2829 | MVRRKSSPTRNKSSNRPDIAEYYKIGNKRYKVNVNTMTEIQRLQNSTANCIPKLPFSRLIRELLQKYTTIDYRVEFRALQALQEAAEIYLTQLFEDSNRCANHAHRITVQPRDMQLALSIRGPNNL |
| CenH3 | COLEOPTERA | *Rhyssomatus lineaticollis* | Locus_2076 | MRKKSSAPRRNTTAEESSTTSITATPSRSEQSLPPRRTETKRKRKAKKKSVYVWFGRHLSASAKMISTIRGLQRTFHDLIPKRPFCRVVREIVADVGYPDYKIQSLALSALQTVAEDYLINLFTDAQKCAVHGKRSTVMPPDFRLIEDLMQSMGRVRAA |
| CenH3 | COLEOPTERA | *Tetraopes tetraophthalmus* | unassembled reads | FKINISILRAIKKLQISTFLCIPRLPFSRLVREILMDINREDHREALRALQEATEMYMTYLFEDANRCAHHAKRVT |
| CenH3 | COLEOPTERA | *Anoplophora glabripennis* | Locus_38392 | MVKRKSIPKKAPQSPGKRRKTQTQMYQIGTKNFKVNSTTLRSIKKLQTTTTLCIPRLPFSRLIREIIMDLYRIDHRIERQALVALQEAAEMYLTYLFEDANRCAHHARRVTLLPKDMRLVLEIKGCSDP |
| CenH3 | COLEOPTERA | *Leptinotarsa decemlineata* | Locus_7707 | MVNRKSTPKRKKAPSKQVTKIFKLHNKNYKINVTTLREIKRLQSTTSNLIPRLPFARVIREILMEHTTSDHRVQLEALQALQEAAELYLVQLFEDANRCAYHAKRVTVKPNDMNLVLEIRGILDPGYS |
| CenH3 | HYMENOPTERA | *Nasonia vitripennis* | NP_001136354 |  |
| CenH3 | HYMENOPTERA | *Bombus impatiens* | XP_003489478 |  |
| CenH3 | HYMENOPTERA | *Apis mellifera* | GB18566 |  |
| CenH3 | HYMENOPTERA | *Atta cephalotes* | ACEP16887 |  |
| CenH3 | BLATTODEA | *Blatella germanica* | Locus_75149 | MVRRKSQAPGQSSGHKKRKSQKNKRKSSSASKTTTDEINFTKRRYRPGTRALMEIRRYQKTTKLLIPRLPFSRVVKEIISYIVPPHLSDFRVQFAALEAIQEAAEMYLVQYFEDSLLCALHAKRVTLMRKDLQLARRIRGRFDSAAMSY |
| CenH3 | ORTHOPTERA | *Acheta domesticus* | Locus_18826 | MVRRKRSSNTLKKIVDESKKKSAVNEKFQRKSTVHAFQIASPEEKHVTREKSAVDKILSDFEENEQNLVDGVIESAEDSEVNEPLPNKSKSIRVKKNANLGVLREIRKFQKSTDLLIPKLSFARVCKEIFLHYGGPDVRVARAALECLQESAEMFLVHLFEDSYLCTLHAQRKTLMNKDIRLARRIGGRSGFYP |
| CenH3 | PHASMATODEA | *Sipyloidea sipylus* | Locus_63869_Transcript_2 | MVRRKSTPRSSSSSSKSTHRSSTTKSSRTSSSAKTKLGQGMKALCEIRHFQKSVGFLIPKSNFKDAVKQTLVNMSHRCSFVNQIRFSKLSMEALQEAAEAYLVTFFEDATLCTIHAGRVTLSVRDFELTRHLRGCTEVGNK |
| CenH3 | EPHEMEROPTERA | *Ephemera danica* | Locus_52524 | MRKKSTPQTNRTEKSGGQNAAKKKGREKQTQSTPKRTMKQSQSTPQKGSVADVKSATHKPHRYKPGNRALLQIRQYQKTYDLLIPKLPFSRLVREMMQLLRPSTDWRIASGALLCLQEAAEAFIVQMFEEVQLCAIHAKRITIKADDIRLVRRLRGPSNVGN |
| CenH3 | MICROSPORIDIA | *Nosema bombyci*s (*C. tenera* assembly) | Locus_9192 | MARTAMTTKNTSGKGKKSIQKEKTKTQPKKSVSVKKPVIVQGTPTRRRKTKHTLVLKEINYYQQSTNFLLSKRPFVRYVRRVVGELAVSKDVSTVKFTAQSLEMLQDIFEAHITALMEAAYNCSRHAKRVTLYPTDLRLVSKIRG |
| CenH3 | FUNGAL | unknown (*P. glaucus* assembly) | Locus_1862_Transcript_3 | MYVDYKKQKTISADAILYALKLNNIKFYKVCKSVHGAKKRDVSDKILKYIKYYHRHTTFYTPFQSVFERITRKIVNKLNESNYRIQPYAMMILKESCENHLFVVMNTSYFATLHRNRVTLSASDINLHQRIHPELDHADVFIHNSKIQDIRTNKLCVNQMKRVDKHKIKKSKISL |
| CenH3 | FUNGAL | unknown (*B. germanica* assembly) | Locus_35695 | MARATSPPIISNSHSPMRKAPSSLRGRAAKSKDKSFTKEVQGTVIDGSAEKMLIPRVRRYRPGTNVTREIRRYQNSTGLLMGRLPFARLVREIAQDLTTGTATELPLRWQSSALTALQE |
| CenH3 | SCORPION | *Mesobuthus martensii* | gi\|553813810\|gb\|AYEL01086472.1:3843-4067(-) | DLLLRKLPFMRLVKEICAELTGCFYYWRTQALLALQEATESYLVNLFEDSYLCSLHAKRVTLMPSDIRLARRIRG |
| CenH3 | TICKS | *Ixodes scapularis* | XP_002409763 |  |
| CenpC | DIPTERA | *Drosophila melanogaster* | FBpp0088911,FBpp0308429 |  |
| CenpC | HYMENOPTERA | *Nasonia vitripennis* | XP_003424350 |  |
| CenpC | HYMENOPTERA | *Bombus impatiens* | XP_003489733 |  |
| CenpC | HYMENOPTERA | *Apis mellifera* | GB43219 |  |
| CenpC | HYMENOPTERA | *Atta cephalotes* | ACEP25354 |  |
| CenpC | BLATTODEA | *Blatella germanica* | Locus_13739_Transcript_1 | MSKEGVMFGDAITARKILMKRMEISMQKSRIASVHNKDVESRDQTLMSPGCAAMPKDSLTLNTSYQALTRIHLDSWLKSLPRWMNETISTAGSDALQNLTYNYSKINVTSTNRDYSSISTSQDRTNLRNKITTSTLVSRLCSEQEKMDVDRTIEECAIGVSSEPAISVANGETNLCNEYEQQMVQEPVVAHKPPKKEVEFKRWGLILTIRGSLFIVDYSTRSIKRSRDISSCERVDAHTMYCSDGTVYRLLGSPNFIDKVPIFVQDKFKSGFPPNWQSIMIKWLQFLKQGKPSDFDWTDSDKTMETLKDCFVKINPLKITHECYDKLRTLEKWTPMLVKPSRLIIKGFLSSSQNTHTTAALKNVQDDKIECEDGAEYKLIGPFIDSCKNIPNEIQKYLQKGMPSKWKEMVAKWEALEMTSSENEKHEVPTDMKLKPLEETTLQRSRRGRIRIPPLKFWLGEKMVRNYDSTMVLICGEDDVQKQLARKNEETGQFQHVLKEKQGDQLKRKNKKIDKTGLSENLGKEKQGELKRKNKIDKTGPLQHAIEEKRSDQLRKSKRNKQSVTAKKRKQDKGKSRRNELPEYEKDEESHTSGKVDNWLAKINNGEEDILPGGYEENSNSVDYDEHFKNLLANKSAVTSCTQSPIHTYMTREETPVSSFIQSSPSRPSFSSKWISQYIQREVDIQKKRKPVEHIDNIQKKGKYEQQTGKYINLQYEEIVDRWFSDSDLSSSS |
| CenpC | PHASMATODEA | *Sipyloidea sipylus* | Locus_16394_Transcript_3 | TDSTKNKKDANSELRLVVRDIFGKSKEKEARIKRASEESEGPRKSKRQRVAPVQYWKGERVQYEPIEHPECGLMYKLVDVARPRVNEATKKIVHSDSGDRLKKRNNAMKKIHQKRNDHDTEDTLSSSKSISAAVNLREKIPIRLNEKTEVVECVSHFSKAALEPFVLHSGESIVGIQYQPCLSLNGFTNGILQMAVGASLKNDINGNVDLLLFVARGKVDVKINDSLIHARPSSSIIIPKGVLYKIKNTSTSETLLFYVTKI |
| CenpC | ODONATA | *Ladona fulva* | Locus_17537_Transcript_1 | LHNPNASQGLTLNNEESSSSTKDDSQHLFPLSPRLVRKRSAESSSDYLRKKVLTMRLGKSICHQSLMENEEQLVTSTPVKPSSSRIALSSSAKIPDGEIVFCGNTGNGESQPTVNEVLERINEETEEDSKKNSTVKTVEEVKGNETNEERKNKDEAEKASSNIKESPSEEKENALTLGIKKPKSPNKPTEKFVVDKILSEWPVDEICASSNEKLKQALTKPNKVKALVNGREQEIETFVEFSQIKFHKHKKQNIQYGPAVNTPTYGSGLVHLTPGSDKGVSVAKQCNFIYVVLGGLVEVKIYNETERLCKPGDFIVIPKGTKYNITGRGDKVSALIYFKCQGTETI |
| CenpC | ODONATA | *Libellula vibrans* | SPAdesNODE_6260 | PNINNRVTYKKDSSQCRGTMRKSVKKGIQTKTSSAMKLKQNLSSSTKLGKNVSRKHCRAQTESSSEDVDGSIGQRQASIISVHAKTPVSRKRSRKSAGETTGVAIKSGKKLGNERGKAGSDSKSSEMTESDGPPEKVKRGRPKSAKSKALEQQRRNPASQEESSDNTDTVGKGKTLAGVGKGETFVSKVNVSKNSKLVPSESTDFETPPVKGKRGRPKVAKSKVGAGQQMKGQLKSQSESSETPESGSPQKKKKRGRPPLKSKKSSDAQSEVQADIKSSDSNSEQLECSEASNVPPKRKRGRTAKVEKTLPAQKKTGELSKQNQSKVHMTETVNGEAPSNEKKMAKGPRKNSKKQKLDPEAEKLEMNNDGLHVSSSDSESDKSGVPKRRPLFSSEKFTNELLLKQDEVLPEPTEKKTDVPPKLSPLRDKKKSSESSSDYLRKKVLTMRLGTSAFLNSVIEENALVVTSTPKKSSAESRTQSTSGGSLSDDVFDRNEGNTSNVDQQATSVRLDGDSEKNDSLNKSQITAEVNKPVMLPVHSNEDVLNQKIQPVDDPVSDNKSPQSSSEQVATEGTSNATKEEPVKKIPDSAPSQSLKVNERLAGVPLTNTATTESVASSSSKSSRTKSSVPVVADGNLVELELFADYSKIPFRSRQKESKANGVQYGAALDVLGLGTGYIHLAPGTEKGLQHPKSFNMAFLILDGQVMVKIHESDIVANPGDFFAVPKGCKYNLTGIGDKNSVVAYFKSKAL |
| CenpC | ODONATA | *Libellula vibrans* | SPAdesNODE_13580 | MPHEGNVSTESRRLQVICSTPKRGPVQRKSRLSQATRSSSRFSGLRSPGISPVSAVSYARENFSDAEDFEEASVHREKRQRPTDLLGNRNRRKEQRNTSQNRVHDLEMKQASRWGASQLQRKIHYVPLVVVSDEEESTRLGKRGLARQNLAKKHAVVTRTVYRNSVRESDSDHESLDSGKGGSETQVSVPAAMNSGSALDNGSHPHTDFASTQKETSVCKILKYDSGMGKQASSNQSILDLISSTQWQSNRPYYEPLVIAVSDDEEDKVVMKKNRGNHKKKPMKCITNNMVAGAGRTRLKELVVNASFDDYEDDKEEDFEEERQQNMSSNETEEDYLDDIHREKCGQSEEEAYSSENESYSSARSHETMGQRRLSRVYKERDKAEDVLNDSFCSEDEDEVLSGSAIFNVNSYVKERGKQFYEQGRHETVVRKSMCPVRRYVYDNEVEDVFEDSGELATELELSGPCADSSCRIRGVQEDVSDEEEIGVWNISGPRNRQSYIPRSQNDVQNSKKPAVRGKPGRRRAVLQSSQTDFDENLDKKSEKPPRKAPQKKRQSLSADDDKGSAKRKRGRQSMVKANEGDPQPSRKPGRKKKTESHDDTCNSPSDKTSDTEAPLPKKARKKINLSPTQAESVADKKPYSRKRKSEDSDPSTKSIQTKKSRNTPKARKSEKGGSKSTADSTEEASKDIVKRKRGRPPSKGKKPKGDDTKQPALEGGKDATVEAENKSTICEDATGKHRSLLNPNASPDGLSLDNGKDSSSNKEDLQHSFPLKPKIVRKRYAESSSDYLRKKVLTMRLGKSICPEPLIENEEQFVTSTPVKPSSSRIALSTSSKEPDAAIALSFGNSGRSESQPTAKVSLEMRISEEMEGDSAKNEKKNDITDVNEEPKSGDVTNETGLNVKDSEESSSQEEVSKTVKESLSEGKENTENSANIQPITPVKHDVKFVANKIITEWPADEICATSNEKLRLAPSKPDTKKVMVNGVETDAFVEYSHIRFHKPKKQNVQYGPAVNTPSLGAGLVYLTPGSNKGVAVAKKCNFVYFVLDGLIEVNLNESEMLFRPGFVSL |
| CenpC | EPHEMEROPTERA | *Ephemera danica* | Locus_26258_Transcript_1 | MVIMIKRKQNQEKVPEEKDDESIIIASTPLNRNLRNVRAQQVVPIQLSPVLDSRALSKNRLKSKNKEEIESKVEVATKTDAATAAKIAEKPASQSLKNPIVNTNRPVELVIDDSDDDVTVVTPKRNVKVAKTKTAPAKSVSKKPVQKNNVVSPPTNPMPAPSKPVPEKLPQTSPLKRPMFEFTIPNKRPRIFIPEDFEDFDFFSDGDPPESEKNPKFELPRPRLNFETTPEQELSQVIAQPGQSTQNFYPKVNDFLVIDDENLHAPIQTGKKEKPTKTSNRSASPKNLKKGITAKQPSAKKTKTKKSDELNAAPESENDGSCDSEALHEEENVPVLAEPMAVNENKPQKTAKKAISQAPKVVFPPPTNAEPVGKRTRSPRKPAAKIQAQTKEVKNTFQSDSSEESDQSQTTVEQVVTSKKRAVDVMDSETEDQAETPLPKTKNKVLRQSNTASQKKTVKNEKISTTPVEDTRTKSRGRRAAQPVSNQQSPKETMEELGVGRRSQRSRVKPVKYWAGEHMLYKEDSKGELEVLQVKLGTDESDKPDTKGRPRKDLYTVGSLLRKLASPADQMEENEDLPPDVDLPTLIHMKLPFNAAQPKKLTEIEVASEVRNLEAISKMEGTSKEDSNIRYSTTLDSSFVSCGYIRINAGSSKGLQISQNMTVFSLLTSSKLVVTINGEKINIGFGQHFWVPPGIAYDIKNLSSLPSLLQFSTVDIGEAYNVLGHKK |
| CenpS | COLEOPTERA | *Tribolium castaneum* | TC005212 |  |
| CenpS | LEPIDOPTERA | *Bombyx mori* | BGIBMGA005251 |  |
| CenpS | LEPIDOPTERA | *Danaus plexippus* | DPOGS214080 |  |
| CenpS | LEPIDOPTERA | *Heliconius melpomene* | scf7180001242448:4159-4230(-) |  |
| CenpS | LEPIDOPTERA | *Plutella xylostella* | PXUG_V1_064474 |  |
| CenpS | LEPIDOPTERA | *Manduca sexta* | Msex000150 |  |
| CenpS | HEMIPTERA | *Rhodnius prolixus* | RPRC006901 |  |
| CenpS | DERMAPTERA | *Forficula auricularia* | Fau_Trans_V01_Contig_21396 |  |
| CenpS | DERMAPTERA | *Anisolabis maritima* | Locus_1026 | MENENPGPSRAEDLSVTEKNQIGLYDAVEKIVNEIENVIQIKFDRNVQTLIAELTWRKLKIYAQDLEAFSMHRKRTTIDIDDVKLLTRRNPSIQEFIKKVEEPKPKRSAKRVKKE |
| CenpS | PHASMATODEA | *Sipyloidea sipylus* | Locus_52504_Transcript_4 | MDKLSHEQKLRATIYYDVRKVCFEVLQNVKFSANRDVVDIVAELVWKKLQIMSQDLELFAKHRERLTVNVDDVKLTLRSLPSLKEIITQMADEQAKKEQAKKKQSNAAPSEAKKELEKCEVQNDKSNF |
| CenpS | ORTHOPTERA | *Acheta domesticus* | Locus_41382,Locus_41383 | MGLGMEKAASDLISELIWKKAKRIAEDLELFAKHAKRSTVNADDIKLLVRHNEKLKEDISKLAEDMKKAKKSKKSNVATDNDFADIDSV |
| CenpS | EPHEMEROPTERA | *Ephemera danica* | Locus_31894 | MDNKFDNLTHEQKLKVSVFNDVRKICEEVGQTTKMSFSRKSMDLISELVWRKLKIYGEDLEAFSKHAKRATINSDDVKLLVRRNASLRAHISKIADEINAGKEPRKGKQQKKEAAPASDAGSSTSTDTGGGGSVVNNRADSPFEEDPFLMDQ |
| CenpX | COLEOPTERA | *Tribolium castaneum* | ChLG7:15781374..15833239 (+) |  |
| CenpX | LEPIDOPTERA | *Bombyx mori* | inferred based on EST | MARNIKDNNNIDPATLLSNVKSTIKKDVIKELLENHFQESKTKIAPHALMLLADVAKCLVTETCLRAVKQAQREGSNKVDVEHIEKCLPQLMLDFP |
| CenpX | LEPIDOPTERA | *Danaus plexippus* | DPOGS214323 |  |
| CenpX | LEPIDOPTERA | *Heliconius melpomene* | HMEL002951-PA |  |
| CenpX | LEPIDOPTERA | *Plutella xylostella* | PXPG_V2_029063 |  |
| CenpX | LEPIDOPTERA | *Manduca sexta* | Msex011371 |  |
| CenpX | HEMIPTERA | *Rhodnius prolixus* | supercontig:RproC1:GL563007:395361- 395576(+) |  |
| CenpX | DERMAPTERA | *Forficula auricularia* | Fau_Trans_V01_Contig_38657 |  |
| CenpX | DERMAPTERA | *Anisolabis maritima* | Locus_55963 | DLSNLSYRINRDLIKNILKEEFCNDKTKINDEALTVMSELSRILIVEASLRASRQSYSENSNIVHLSHFEK |
| CenpX | PHASMATODEA | *Sipyloidea sipylus* | Locus_5632 | MTIPKITSKIRHETVKEVMKMHFSDTKTRVCDDVATLVSEVCKNLAVEATLRACQQASRQNSTNVEMEHVQKILPQLLLDFK |
| CenpX | ORTHOPTERA | *Acheta domesticus* | Locus_24109 | DDPCADAFHTEALKQILKHHFKDPKTRASDDTARMTAHVLEIMAREATLRAGKVAEASND |
| CenpX | BLATTODEA | *Blatella germanica* | Locus_10933_Transcript_3 | MPPGDFNIKKLTSNFKLEAVKEIMKLHFSDSKTRLSDDTLLLVMQVLHTLTVEATLRAGKQARLEESTRIQLEHVEKILPQLMLDFV |
| CenpX | EPHEMEROPTERA | *Ephemera danica* | Locus_17019 | QFKAEFKPELARDVSRLGCQDPTKTRMNDDAARLLAEVTRLLTIEAALRSCQHASRLGGDEVDVEHVEKSILQLMLDF |
| CenpI | HYMENOPTERA | *Nasonia vitripennis* | XP_001604088 |  |
| CenpI | HYMENOPTERA | *Bombus impatiens* | BIMP24574 |  |
| CenpI | HYMENOPTERA | *Apis mellifera* | GB45366 |  |
| CenpI | HYMENOPTERA | *Atta cephalotes* | ACEP26914 |  |
| CenpI | LEPIDOPTERA | *Bombyx mori* | inferred based on EST | MCDIGEIIDYIKSLKRGFDKDLFQSKIDELGYIVDSVGLDYDDFHTLFKIWLNLSIPLTKWTSLGACLIPPDAVEEKTVDYAIQWILINYGNQNSFTKTGFLLDWLTAAMECDCIDMKALDFGYELFYSMMTYETLTPQAVKLVYTLTKPSDVTRRRVIEILDCAKKRESKKNLFRQLQILLGLFKSYKPECVPENVPAICVHTAFKKINKDLLGRFKRTQERRNRQSKEKQHLIWLNPINMARGRNKKIVPLVPNMEFFNIGSKQYDQKEPQKNYLDFSDPASVVQLAACGRAARPARLRALLCGPALLLAAAAADQHAFLSHDLRHLLDNCFLDISPYSYAEKQDLLQRLALLQSTLLQGIPVITRFLAQFLPLWNERDYFTEILQLVEWVSVDSPDHLGYIVGPLIKIYHRAQPLEQCAILKSLTQMYINLVYASTRRRQFFMAIDTPKENYDVVLPRLAAELGEMGEKALQYNPDDMRVLFSNIWSVECRSRAHLLYNIGLGPIPGLLSLSLPLLGVSAALIEKMAALLVIYKKIFTRLKSTNAISATHTEQIQILHRYSVDLSSLYTEESLRGRGNGFVFDKLHPQLVSKLYHLIPEPDTKLSVRSHVAFAAYTYVALGDVDERDADNKAWYRAFLEHEFSYLAKFFKKTVPELRM |
| CenpI | LEPIDOPTERA | *Danaus plexippus* | DPOGS201324 |  |
| CenpI | LEPIDOPTERA | *Heliconius melpomene* | HMEL012411-PA |  |
| CenpI | LEPIDOPTERA | *Plutella xylostella* | PXGS_V2_008864,PXUG_V1_012553 |  |
| CenpI | LEPIDOPTERA | *Manduca sexta* | Msex006319 |  |
| CenpI | HEMIPTERA | *Acyrtosiphon pisum* | XP_001952498 |  |
| CenpI | HEMIPTERA | *Rhodnius prolixus* | RPRC004146 |  |
| CenpI | PHTHIRAPTERA | *Pediculus humanus corporis* | XP_002428653 |  |
| CenpI | DERMAPTERA | *Forficula auricularia* | Fau_Trans_V01_Contig_4938 |  |
| CenpI | PHASMATODEA | *Sipyloidea sipylus* | Locus_30911 | QQQSRFSYLLFNKLYDALILKEPFPRDEIEDCLRYLVELEEYMQQGVPVVSKILSEY |
| CenpI | ORTHOPTERA | *Acheta domesticus* | Locus_18813, Locus_22087, Locus_25057 | PGSFICLQWVAGLLEFGLVNEKPVNEYYYMFHILLEWPRTEKIACWLVVRLTKPDDIDRNMVVKLINDVKANKSVRDIILWCYKTYKPECVPEAIAARSVKPTLRLSAYYKYHFGMSLQRISADESFKLTHDRQTDLDVIWTIPERKKRRVEDVIPGVEYISFGSKLYQEKRVSILQINSWEKFATLALKIDIPCNALSLIQNPIGYHVLACGNEDLQTRFSYTLLHLLQTVVIDRLVKVPLEKQEYLLEKVCEFQEYMQQGIPVMSKFVAFYLVHWDGFTFLAQLMRLLPWITFSSFNELKNLYLVHLYYVYISSNIMVKCSILRALLQLAQNLILQSCGVVERLFLHSPVLENSE---ENSEEILYKLIKYIESLCILGLNFEPDSAVLLHEVLRLYEILLDSEERSGLNIRSVLPRAAVYFSLFSRNYGNIERVCGLLLKYDASIGIQKSLEIKVSGRAKLVKRYSCDYMNCLWLMQAFSGREEGFIFKGVEPGAFAHVENLDKAFNIMGCIPLI |
| CenpI | ODONATA | *Ladona fulva* | Locus_6081 | MKCLIPKTYVPDQVVIDVAISILSYIPDQPTVKVLCAVQWLIGVIGLGLTKTKKLDCMYSLFFDLLLKTNIDLQLCPLIQLMTKSHLISRKHIHIVNYIRKYKGKKKVHDSLLSIFKRMDPDLVPEGLPPPLPTQSLVSGLAESWKGLHMAKSRIAEQFGMRMNSTVVQSSHWDKASISIIPQISYLSPKESRKRTLHDVASWEDLGTNMSYLNPPCQSMSLLRSHFGMFFLAFGSHCLQKRFSSSLHEILLAVFVRKQHNIFIKPEMREAGEKHLLQQVAVFQEFLGQPIPVVSAFLAKYLQMWSGDEHRVLLLRLLCYVHFSTFDELKSVIITPLSMLFISGDLEFKCAIINMFKEFMKNLMLKEKCREGKKSPFLNLTRTWDPADVYPKLFEEISYLISIGLSAEIGKSLFISTCLAYYHTMLSILCSLNCEYMLVPPPTLMYHSLFSHSLQCLSQACMLLIRYKNEVFYSSDATKRREKFKDEFSILGVVTEDYLRFLCSDEPFKNRRRSFILKYLSDEILTSLFTNFNLENYMNICYHPAFALHFLSFKKENLPITYKAFYLLTEKKYPGITTFIKDFGPTRNL |
| CenpI | ODONATA | *Libellula vibrans* | Locus_62915 | RKRTLHEVESWADLGANMSYLIPPCQSMSLLRSRFGMFFLAFGSRCLHERFSSNLYHTLLSVFVRKQHDMSPEMREDGEKYLLQRIAVFQEFLGQPIP |
| CenpI | EPHEMEROPTERA | *Ephemera danica* | Locus_103060 | METKVKILLSVGNLILNLFWRAKKIESESTADAEQFTLTSCCKFYERLFNIAVASNKPNNCLNLIATALHFYKMVYQLESHLIKVKVWALPPPTLLFACLTSSHCWLVNSACDLLLRHREKAIRVQREQKV |
| CenpL | HYMENOPTERA | *Nasonia vitripennis* | XP_001605964 |  |
| CenpL | HYMENOPTERA | *Bombus impatiens* | BIMP13555 |  |
| CenpL | HYMENOPTERA | *Apis mellifera* | GB51540 |  |
| CenpL | HYMENOPTERA | *Atta cephalotes* | ACEP26533 |  |
| CenpL | LEPIDOPTERA | *Bombyx mori* | BGIBMGA001894 |  |
| CenpL | LEPIDOPTERA | *Danaus plexippus* | DPOGS207530 |  |
| CenpL | LEPIDOPTERA | *Heliconius melpomene* | HMEL017958-PA, HMEL013522-PA |  |
| CenpL | LEPIDOPTERA | *Plutella xylostella* | PXPG_V2_022199 |  |
| CenpL | LEPIDOPTERA | *Manduca sexta* | Msex006101 |  |
| CenpL | HEMIPTERA | *Acyrtosiphon pisum* | XP_001951878 |  |
| CenpL | DERMAPTERA | *Forficula auricularia* | Fau_Trans_V01_Contig_16134 |  |
| CenpL | PHASMATODEA | *Sipyloidea sipylus* | Locus_11805_Transcript_2 | KTFKRCGLLNGSFKSCSISPLNNFDYSEVCLKSYSKKLVQHLTNVHPSSSSAHFRVEFATEEGLAQTSHDNTAIRITVLYKVNGQEKTFYKGLLLSWNCLRPVPFDGCVYLPLLLYWGSKGAGKLVHDMLAKYFDCVISGLTIDHKDLICLYIAIANKFDTGRRSQWLELHYELPSCPPSEQIVICLPIEQQKELWRCILNADKSSIDIGEVETYYKCMMKHILRTYSINVGSLRLVGIKVSTNATVSTEAKVKVRSPEAAVVVLNLLSDLSTRLESARAPMSQLDPCLASMY |
| CenpL | ORTHOPTERA | *Acheta domesticus* | Locus_11531 | YLGFFMSWGARRFPSGCLFLPLLLCKAKTIKIKTNIHAEFRKLFDCVIEPYQLKHHQLVLLHCLFVNVSKGARDEQKNVVRYTLLPTWANKDKIEVFVPLPDLRKIWTKIRSK |
| CenpL | BLATTODEA | *Blatella germanica* | Locus_46581 | MPNKHVHSPDVGLRTRNVEETDITSTNQYTPRPERLRFSYTNVSESDDEELDVLINRRWKVFRVSPLNRFSYTEQRLKQYARRLQEGLATLATGSENAKYDVKFGVESGLTQTRHDKQAVKISVTLVSRTGDDGDGDDEDSGEIPPKLFYLGYLVCRGASRSLEDVSMTHLPLLLCCGTVMSLRNINFLIQKFFDCFISPLELSQDDLIWISAIIAQEEGHTVEFNFKFSQITVKNAISCKMDHTDLQTLWKAIHDPE |
| CenpL | ODONATA | *Ladona fulva* | Locus_30011 | SCSYGVNLHECILLKVMMPNASFTTDGKLKISSLILAGRILRFLADIC |
| CenpL | ODONATA | *Libellula vibrans* | Locus_44856 | MAWLVCLASSDPRESCDEKSALKEVSFEYDLRRTETEKVKPSVIVSHELGLIHQVWDSVHRNFGAEDEVLPEEVSIFSGCIAEHSFCSYGVNLHECVLLKVTMPSASFTNDGKLKISSLILAGRILRFLADLCAVENSVTPTLGFSVTIKHSVSDR |
| CenpL | EPHEMEROPTERA | *Ephemera danica* | Locus_12841_Transcript_4 | SQSVTFYLISDVVDKKTWELFCVSRLFNFEPSEEKLRFYNKKLREMVAANLKHDMVAKIHAEIAVINEFATTTFDHEAISIEVQVQEIQTDEQRKPRVFIYKGYMLSRGKLNSQDVEPQLPLLMCRSKNINLISSVHMCLAALFDCRVSKLELRQVDLQWILAILLENDPEIKDQCIDFTLTYQPHTSPKDKVSIGMSALALQKIWRCIRSKNIKETEADFDEVALLFSSVGQHIQATFNLQVEHMGLTGLSSTFGNFLSTGKVIFTSMEVAEVILRFLTNQLLLEEKFGVELFSKQERMEACNT |
| CenpM | HYMENOPTERA | *Nasonia vitripennis* | Nvit_2.0\|Chr2.Scaffold5_3422046-3422513(+) |  |
| CenpM | HYMENOPTERA | *Bombus impatiens* | BIMP16067 |  |
| CenpM | HYMENOPTERA | *Apis mellifera* | GB50848 |  |
| CenpM | HYMENOPTERA | *Atta cephalotes* | ACEP13173 |  |
| CenpM | LEPIDOPTERA | *Bombyx mori* | BGIBMGA008546 |  |
| CenpM | LEPIDOPTERA | *Danaus plexippus* | DPOGS202632 |  |
| CenpM | LEPIDOPTERA | *Heliconius melpomene* | HMEL010219-PA |  |
| CenpM | LEPIDOPTERA | *Plutella xylostella* | PXUG_V1_030634 |  |
| CenpM | LEPIDOPTERA | *Manduca sexta* | CUFF.3181.2:6812-7351(+) |  |
| CenpM | HEMIPTERA | *Acyrtosiphon pisum* | NP_001233082 |  |
| CenpM | PHTHIRAPTERA | *Pediculus humanus corporis* | XP_002422917 |  |
| CenpM | DERMAPTERA | *Forficula auricularia* | Fau_Trans_V01_Contig_34953 |  |
| CenpM | DERMAPTERA | *Anisolabis maritima* | Locus_30076 | ISILIVSGISSMAEQLLQSFGEMNDEISVSCCDSISSFLTNIDDNHLPFDFIIFIVDTRSQVALVELEDNIRLLDPTYLMNRLCFLQPKISLDKM |
| CenpM | BLATTODEA | *Blatella germanica* | Locus_55919 | MAGVLKSYGNAEVKPDCVYILVVASTDMCVKLSKALHIASRNQGVNLQVHICNKIQDLLDSDLHSQINFVVFGIDARNLNCLNKVKDNIKQLENFLKFGRMCFVNGYNVKPAEMAVTYNSIWELSIAYNVHLIKGNVEVESKCLYLADRILRLAALTVGVKTGIPFIECPFRGTP |
| CenpM | ORTHOPTERA | *Acheta domesticus* | Locus_32343_Transcript_3 | MTLSSRILNLSVLVVGKNPELRDLQHSLQWQRRKMNIKGDVKIDLCEVISDVLSTEKAIYHDFVVLTFDMRMYEIDEIRSALKKLKKQVLLHRVCLLSVVDVPSCEQRIDYDDISKCTEKYGIPCIFGDIRCKVKCLYLAERILTLVSATMGLHTGRPTIF |
| CenpM | ODONATA | *Ladona fulva* | Locus_16742 | TTRRTFFISSDTLNLDYGFFELYKKYKCPAVVGDILEPKGCASIARRALKLIEFSGGMNSGLPFLGLD |
| CenpM | ODONATA | *Libellula vibrans* | Locus_55071 | LTCKRIAFVSGVARMTEHNAWDLRRKYNCPALVGNILDPKACECLARRILKLVEFTLGSATGLPNITYKETYEDLGNKSCLKK |
| CenpM | EPHEMEROPTERA | *Ephemera danica* | Locus_37868 | MANSGLPVLNFVGRNFSQNGLSILIVSRASSAITRNLCQTLQDCRSSSILLHVFTTQSIKQLLQQTKSMSFDFVVFLIKSGRNKGLQELNDDICMIDKHYFTMSRVCLVLYKDVPFSRMQISSEEVHCLAATLHTPVLVSHLENDEEFRVLANQILNLSMTVTGAASGVPLMLAHSQEERDWLSEYFSALY |
| CenpN | HYMENOPTERA | *Nasonia vitripennis* | XP_003424999 |  |
| CenpN | HYMENOPTERA | *Bombus impatiens* | BIMP14985 |  |
| CenpN | HYMENOPTERA | *Apis mellifera* | XP_006563575 |  |
| CenpN | HYMENOPTERA | *Atta cephalotes* | ACEP14227 |  |
| CenpN | LEPIDOPTERA | *Bombyx mori* | BGIBMGA001788 |  |
| CenpN | LEPIDOPTERA | *Danaus plexippus* | EHJ73557 |  |
| CenpN | LEPIDOPTERA | *Heliconius melpomene* | HMEL010819-PA |  |
| CenpN | LEPIDOPTERA | *Plutella xylostella* | PXPG_V2_023081 |  |
| CenpN | LEPIDOPTERA | *Manduca sexta* | Msex011812 |  |
| CenpN | HEMIPTERA | *Acyrtosiphon pisum* | ACYPI007356 |  |
| CenpN | HEMIPTERA | *Rhodnius prolixus* | RPRC010798 |  |
| CenpN | DERMAPTERA | *Forficula auricularia* | Fau_Trans_V01_Contig_41376, Fau_Trans_V01_Contig_24250 |  |
| CenpN | DERMAPTERA | *Anisolabis maritima* | Locus_23680 | TLEENFLEPPKFVPDPPKFVESLNGVIINQDEARKKYADDIFGETPPTLDSFTIMSTSKDPQKHYPFYKGENLVDIRLKFTSRNVMDTYKKCIKNKIFNVPLPDYLNNIPTSGKNFIVTSID |
| CenpN | PHASMATODEA | *Sipyloidea sipylus* | Locus_54939 | PLSSTKCLTKTVLGFNKVKKCLLTGYSVSSLFRLLKKQEQNASSWRDVDDIVLRRNLSNIEVVESARGLDFSQSTRRKEYAKNCIGEDPPVLKSFVLKSNCNKWKGGSSVPHFLGRHFETVLMIKSKNIAHTVERLIAQGTI |
| CenpN | ORTHOPTERA | *Acheta domesticus* | Locus_10430 | GVASLSVPSYKVENPRTVFGGRDYTQHENRKKFAAKCFPVDAKLETLTFINGNSKWKDDIGTEDGEDLVLRWVVKISSPNVFSTMKGLCENNVIKPEHAASYALRLGKEG |
| CenpN | BLATTODEA | *Blatella germanica* | Locus_62646 | GTKSLDWLKKNLSTLRLLDVALHNQKLKWSAYRLKKNDGHIYKNCSRIEETIQTRIVNLQKIMDVDVIAHECLYWISMIETIRIPGRGATVKPPMFVGYVVGQPYFFITQNGRKEVFLNVISKSLGYKIFEDCNL |
| CenpN | ODONATA | *Ladona fulva* | Locus_7370 | NCLLSEGGLKRDLTATFEKLNLKYDFAVAMFDEVAWIRVALPSSKADTALYIAYFIGEGFIFVSKRKINFKLMKALSSGLKYESLKVLDLNGKDVWSLLKILKQRKSKALEIDHARCYNQEEYLNNSRVVDFSQKSARRAEIKDCFANNSPDLQSFTIKSESGLRVAEVLPEMSGYPCRSKIRISHPNLHEFLIEASQRNVITQPLPQYMKHFFLTGKNVITLKDSASKK |
| CenpN | ODONATA | *Libellula vibrans* | Locus_65098 | MFEVMDYDYLKEKLAHMQLADVFLHSKKRTWYSFELCGKAHNLMLSEGSLKRSFAANFEKMNLKHDIAVSMFEEVTWIRVGLPSSSTENAIYIAYFIGEGYIFISKRRINLKLMQAFSAALRYESLKILDLNGKDVWNLLKILKQRKSKALDLEFDEDRCHRLEENIHNSRVIDFCRKRPNDTIVNKCLAVNSPQLQSFTIKSESGLRVRGILPEMDGYPCRSKIRINHPNLQEFLKEAIERNVVTEPLPLYMKHLFLTGKNVITLKDSATKK |
| CenpN | EPHEMEROPTERA | *Ephemera danica* | Locus_18529 | ITKGSEERSKDYAPFYMAACLNEPYLFVPHKRRLLTENIFKCVATSLGYDTCKLMTFTGPNITELLHSLRQSQGRAAGLARIANQPFVTGPPELLPGGGLDFTQSKARERYVNEHFGEKPPLKPSITFHTCNDFQSHQLRELDDIEFPVSVTVSSAEGDAWGAVKRLVTEEVISFPPPAFYHQAMLFNRDVIKLRPNH |
| Mis12 | DIPTERA | *Drosophila melanogaster* | FBpp0076645 |  |
| Mis12 | LEPIDOPTERA | *Bombyx mori* | inferred based on Race analyses | MIRTLPWSGGTDEEYETQLFSFGAQRLKIATRQMIEQKITLGIKDMEAYLRESLDLNETDKSTLTKACDKLVRLYCERASPSLEVIDSEIERILKVPENVLLPGDECQVEQMTDSNYAQLKDEVALLRKRVERGALMEALLTAEEEELCTVEKVCESAKKDMEALDLVFKNADNSESLKQIQNETRFLCASTSFMKENDNNIF |
| Mis12 | LEPIDOPTERA | *Danaus plexippus* | DPOGS208427 |  |
| Mis12 | LEPIDOPTERA | *Heliconius melpomene* | scf7180001249512:35940-36548(-) |  |
| Mis12 | LEPIDOPTERA | *Plutella xylostella* | PXUG_V1_009553 |  |
| Mis12 | LEPIDOPTERA | *Manduca sexta* | Msex010766 |  |
| Mis12 | HYMENOPTERA | *Nasonia vitripennis* | XP_003425747 |  |
| Mis12 | HYMENOPTERA | *Bombus impatiens* | BIMP16426 |  |
| Mis12 | HYMENOPTERA | *Apis mellifera* | GB52506 |  |
| Mis12 | HYMENOPTERA | *Atta cephalotes* | ACEP21582 |  |
| Mis12 | DERMAPTERA | *Forficula auricularia* | Fau_Trans_V01_Singleton_51901 |  |
| Mis12 | DERMAPTERA | *Anisolabis maritima* | Locus_29798 | PATLVEAMPDKKEGLQRACAQMTESYIDQSKPYVDQFHEIMLKKATIPDNILLPEDHDQLTQYTEDDEKALDEKIEQLTNEYQRLCVLEEAYEGELKNGENL |
| Mis12 | BLATTODEA | *Blatella germanica* | Locus_32046 | MESNNTRSIKEYEKEAYEMQQFNFSARQVSEYMSSIIEDCTQMKVEDLKCCLLKHCVNISPASIESATAMLLKERHSMLEKFMKQISIAVEEFFGIPANAVLEEDSLQMTQFTKIDEAQIDKEIVLLQERTKRAFILEECLKRERDILCKVKASEMMQKDSLEPDLQLTKNKMRCVIDIMEATRTTGKECFGSSFKICDNAAKRFQCP |
| Mis12 | ORTHOPTERA | *Acheta domesticus* | Locus_38450 | MSTVGAAERQHAQEEYGAQLHKWNPYQLYNNVVEIMKVVVEEGMDGWEHSLKKFVPECNNAIVRSTLKSELFASVDKNGPELMNAIKESLSIPRTFLLPEDEVQKKQYSDQELQDFRKNTEDLLKRYQYNEQLINVLEAEHKILEKVDGIDTNVAEWQEINKKCWEEIAVMKEQVNLLKSKNEHGFNSPQTSTIFNSVQLDD |
| Mis12 | PHASMATODEA | *Sipyloidea sipylus* | Locus_3869_Transcript_2 | MFCNLQIPAMEGEHLCLKKLTELEYETQCFGFSSQTIIDVVRGVMTDAVNSAVDKVAKTITEHMKQSDPEEVKIAANLLLKEYEAAIKESLHTWQEAIEKHFQIPSHVLLEEDEPQRQQYTEAEVKDLDDEVSSLECRAKRAALLDSFFSAQLEMIRETRQRLQACEVRLADEGTTQRLVQQARQLTECTRAFTGRVTALGARELPQALREDVANILRPRFQHLK |
| Mis12 | ODONATA | *Ladona fulva* | Locus_23777 | MDITEEFLRDKRKEEYQYQHFQFTTSTFKNELKDMEMSIIEDAVEKMEKSLLGKYPSKAPVIQSATKKLIEEYAQKTGEWVSSIEGKLESTFIGIPSHVLLPEDKPQVKQYTKEYIEQIQAETKVLEKKLQQSKFMKVKLKEALSASEKCLEAISRVQDGPLQEESMKYLELTTNVLSDLMKINEGSNYIAEKLMDNSSSHEEVPSLQEKNLRMLKELC |
| Mis12 | ODONATA | *Libellula vibrans* | Locus_5656_Transcript_2 | MVMAIIEDALGKMEKSLFGKYSSKTRVIQNATKKLIEEFGEKTDEWISSVEEKLESKFLGIPNHVLLPEDEPQEKQYSREYVEQILAEMKVLEKKLQQGIFMKAKIKQALSSSEKCLKATSKVLHESSWKGSTNYVEQTSEILSDVAKINGE |
| Mis12 | EPHEMEROPTERA | *Ephemera danica* | Locus_32263 | MSHPNKHPGNHNLRTREEYETQFFGSSSTEYSKEVSHIFVESIKSGFEGLSNYMCHIKAKQSPENKASDEDVAKIKAVTDTCFEEFISTISQRLQCLEEYVCKKYFNIPDHVVLTEDQIQTKLTDPSETARLDEEINSMLNRLAALKYQESVLTAELEVGQNTIPKIQQLVEKSEKLALQLKQLPDLQQLVTST |
| Ndc80 | DIPTERA | *Drosophila melanogaster* | FBpp0073681 |  |
| Ndc80 | LEPIDOPTERA | *Bombyx mori* | BGIBMGA008679 |  |
| Ndc80 | LEPIDOPTERA | *Danaus plexippus* | DPOGS215199 |  |
| Ndc80 | LEPIDOPTERA | *Heliconius melpomene* | HMEL003868 |  |
| Ndc80 | LEPIDOPTERA | *Plutella xylostella* | PXUG_V1_008933 |  |
| Ndc80 | LEPIDOPTERA | *Manduca sexta* | Msex006828 |  |
| Ndc80 | HYMENOPTERA | *Nasonia vitripennis* | XP_003424937 |  |
| Ndc80 | HYMENOPTERA | *Bombus impatiens* | XP_003489053 |  |
| Ndc80 | HYMENOPTERA | *Apis mellifera* | XP_003250472 |  |
| Ndc80 | HYMENOPTERA | *Atta cephalotes* | ACEP12324 |  |
| Ndc80 | COLEOPTERA | *Tribolium castaneum* | TC011249 |  |
| Ndc80 | HEMIPTERA | *Acyrtosiphon pisum* | XP_001946571 |  |
| Ndc80 | HEMIPTERA | *Rhodnius prolixus* | supercontig:RproC1:GL554110:158449-158622(+) |  |
| Ndc80 | PHTHIRAPTERA | *Pediculus humanus corporis* | XP_002427721 |  |
| Ndc80 | DERMAPTERA | *Forficula auricularia* | Fau_Trans_V01_Contig_3945 |  |
| Ndc80 | DERMAPTERA | *Anisolabis maritima* | Locus_26079 | CKALQTNQYIEMMNYLLGQFFKKLSLNKHNYINLIIELMKKLNYPGVLNKSWLQTAYSANSWP |
| Ndc80 | ORTHOPTERA | *Acheta domesticus* | Locus_19903 | MQRKQWSYGQGRRSSAPKRFSVAFTEAEDRNKNVGDGKKSFIPRPTNVSSRSSSVEREGLQSSSRSAQKFVRSTSAECIADENVRSVCRSRMTESAFTPKHLVKTGCRNTVSVQRTVRKDTRPLSDRAFQMAELAKVEEYLVIKEIITPGTSLSNSMTLKLFVHIMNSLFDMLFSKQQLTLSNYCELIPSLMRRIKYPGNITRSWLVTANSSHSWHNVLGLLSWL |
| Ndc80 | ODONATA | *Ladona fulva* | Locus_5526 | MDPLTRMRNKFSMGRRSSVDPVRIQMDESSFLNTADKRNKSGIPKFVNSTTRKRRSSSCDRQAWRPAKNQGANNLAGLLFTPQHAVTPRVSLSARAAKDCISQLSCKGKPGRKDQRNISDKSVQLALCRHVCSYLNNVGLDADIFTGGKAPAGPIDPLNMKNIDKNAFINLFNFLFKEIGGKGRIEVNSSNYTEKILFTAKKLSYPGIINKSHLQTPNYPQSWPYIIAFLAWMVDLALAEKFYENVINDDSNVLSIFLNFHLASYSAFNRKEIAGPPPQEDIDNLQKLFCEMHGVPEELMQRLRNSRDKIKSQLEAELANEKELEDGQHNKKQQIRQLSKEIEEFTERIKTVRMKTESLSKEREDVSRGKEVLLAKLCELREEKSKLAMACSQQKMSKTEWDTLASKKRELVEDIRMISEVSSNIKKTLDETDLKITSKKNKLNKQWMEFVNKTIPIQPYNVTIQHLLDQIESFNPLNSDAIQCFKDIEDALQHGLTEIIANVSHLNNQARCLQDEQHEWSLKRKELQKEAELLEKNFEERKNKLVIMKEDFIMKMDELNRIKVEIEAETKMINEQPMRDNKEVLEELKAMKIKKTEDLVKFKKESEDYFKMMMEEATAFYNTNH |
| Ndc80 | ODONATA | *Libellula vibrans* | Locus_87233 | MDPLARMRSKFSMGRRSSVDPVRIVMDESSFLNTAEKRNKSGIPKYVVNSTVKKRRSSSCDRQAWRTVKSQDPSNLAGLLFTPQHAVTPRVSLSARAAKDCISQLSCKAKPGRKDQRNISDKSVQLTLCRHVCSYLNNVGLDADIFTGGKAPSGPIEPLSLKNVDKNVFINLFSFLFKEIGGKGRMEVNSSNYTEKIPFIAKSLSYPGILNKSHLQTPNYPQSWPYIIAFLAWMVDLALARKFYNNLINENNIVSLTFNFHLTSYNAFNRKEIDGPPPQEELDNLRKCFCEILDVPDELMQGLKNSRDRLKSQLDAEMANEKELDDAQLNKREQIRQLSKEVEGFTERIKAVRVKTESIFRESEDVARKKDILLAKLAELREEKSNLAVMCSQQKMSKTEWDTLASRKKELEEDLRMISEVSHNIKTTLDCTDIKITSKKTRLNKQWMEFVNKTIPIQPTNTTIRNLLNQIENFNPLNSDAIQRFKEIEDSLQHALSEVMANVSQLENQARSLQDEQHEWSLKKKTLEKEMETLGKNLEEKKHEFNTKKDNYEMKMNELKRIKVEIEEETKRINEQPMRDNKEVLEELKAKKNKKFEELIAFKKEAEVTFKLMLEEACAFYKINQEKINGFKREVQELLHSRC |
| Ndc80 | EPHEMEROPTERA | *Ephemera danica* | Locus_39154 | MNKRKDSLGTTGSNVNPKRKSSLGLRNWMSSADDRLNDAPSTSRPQPMQRTYSNLGMHKGNDFPSSSRIPRLPPPSYSSRRSSSQEPPTYRKRLNLLSPGSDMQHMSNVGVLLTPVRSMSSFSPSTLSVASSARNSSARRISQKLVVSGPIKMKDERPVNDKLFQANAMNQIQSFMRSHSDGGQSHKLKPMSNTALIATFNTIVSELDPNMRVSKEDYVAKIPSILNSLQYPVSITKSSYVAVGSGTATQQLIAMLAWLVDSVTALSNINPDMCLFPNADLNKEEKILKGSYLTLFDAYGQFKQGGSVTEATLLDLFCKHTGDSLDKECQIQTDIEELKIRLEAQKTDPVREANEGKLQQIAILEEEVNTFQKQYDTNSDNLKSLKSKVAQLRETVKQLDVDLGRLDDQAAELQDQVEKQSMKSAERDSIRTQCGIMRENIAEKHRYQEKLQELIGKVDIDIANTRKKVGNITQDINCLVLEKGLGESLKWKHNPLCSDTANEYLLSMQQLWSSMYQRINMELEDKQATLKAGHQQVAKLKEDLQRKKLQLESKKEEIRRLGAEMITIQLESTELEQKLCEDIEKEKAACCKLQELLNHKPKLAHVVECIKAILDKKQALAEEFEKYKVFVESDLLPSILKMFGDWQVKMGEELLEVKESHTLHIQEIFS |
| Nnf1 | LEPIDOPTERA | *Bombyx mori* | XP_004928275 |  |
| Nnf1 | LEPIDOPTERA | *Plutella xylostella* | PXGS_V2_029990 |  |
| Nnf1 | LEPIDOPTERA | *Heliconius melpomene* | HMEL012303 |  |
| Nnf1 | LEPIDOPTERA | *Manduca sexta* | Msex000636 |  |
| Nnf1 | LEPIDOPTERA | *Danaus plexippus* | DPOGS206630 |  |
| Nnf1 | HYMENOPTERA | *Nasonia vitripennis* | XP_003424749 |  |
| Nnf1 | HYMENOPTERA | *Bombus impatiens* | XP_003488841 |  |
| Nnf1 | HYMENOPTERA | *Apis mellifera* | XP_392538 |  |
| Nnf1 | HYMENOPTERA | *Atta cephalotes* | ACEP17450 |  |
| Dsn1 | LEPIDOPTERA | *Bombyx mori* | XP_004928630 |  |
| Dsn1 | LEPIDOPTERA | *Heliconius melpomene* | HMEL010327, HMEL017423 |  |
| Dsn1 | LEPIDOPTERA | *Plutella xylostella* | PXGS_V2_006948 |  |
| Dsn1 | LEPIDOPTERA | *Manduca sexta* | Msex015437 |  |
| Dsn1 | HYMENOPTERA | *Nasonia vitripennis* | XP_008212482 |  |
| Dsn1 | HYMENOPTERA | *Bombus impatiens* | XP_003490867 |  |
| Dsn1 | HYMENOPTERA | *Apis mellifera* | XP_006558067 |  |
| Dsn1 | HYMENOPTERA | *Atta cephalotes* | ACEP12572 |  |
| Nuf2 | PHTHIRAPTERA | *Pediculus humanus corporis* | XP_002423715 |  |
| Nuf2 | HYMENOPTERA | *Nasonia vitripennis* | XP_003427097 |  |
| Nuf2 | HYMENOPTERA | *Bombus impatiens* | BIMP23012 |  |
| Nuf2 | HYMENOPTERA | *Apis mellifera* | XP_003251494 |  |
| Nuf2 | HYMENOPTERA | *Atta cephalotes* | ACEP12691 |  |
| Spc25 | DIPTERA | *Drosophila melanogaster* | FBgn0087021 |  |
| Spc25 | LEPIDOPTERA | *Bombyx mori* | NP_001040214 |  |
| Spc25 | LEPIDOPTERA | *Plutella xylostella* | PXUG_V1_027073, PXUG_V1_081401 |  |
| Spc25 | LEPIDOPTERA | *Heliconius melpomene* | HMEL017251 |  |
| Spc25 | LEPIDOPTERA | *Manduca sexta* | Msex016135 |  |
| Spc25 | LEPIDOPTERA | *Danaus plexippus* | DPOGS215199 |  |
| Spc25 | HYMENOPTERA | *Nasonia vitripennis* | Nvit_2.0\|Chr4.Scaffold35:132052-131828(-) |  |
| Spc25 | HYMENOPTERA | *Bombus impatiens* | XP_003485975 |  |
| Spc25 | HYMENOPTERA | *Apis mellifera* | XP_393701 |  |
| Spc25 | HYMENOPTERA | *Atta cephalotes* | ACEP12420 |  |
| Spc25 | COLEOPTERA | *Tribolium castaneum* | TC013119 |  |
| Spc25 | HEMIPTERA | *Acyrtosiphon pisum* | XP_001950753 |  |
| Spc25 | HEMIPTERA | *Rhodnius prolixus* | RPRC004798 |  |
| Scp25 | PHTHIRAPTERA | *Pediculus humanus corporis* | XP_002424779 |  |
| Scp25 | DERMAPTERA | *Forficula auricularia* | Fau_Trans_V01_Contig_22160 |  |
| Scp25 | DERMAPTERA | *Anisolabis maritima* | Locus_3149 | MSSKQNKDNQDKPVKVNKWDGSAVKNALDDAVKEVLTKKFNYVENFTLIDGRLAMCSIAVGVAMFALLWDYLYPFPHSKPVLILCVSTYFVMMGILTLYTTYREKGIFVVTIQKDPAGFNPDNTWEASSYMIKYDDKYNLILNY |
| Spc25 | BLATTODEA | *Blatella germanica* | Locus245_transcript_6 | QDTVIGLQALAKLGEKMSSSVTDITVQFSYPGGNAKPIKINKWDGSAIKNALDDAVKEVLTKKFNYVENFALMDGRLVMCGIAVGVAMFALLWDYLYPFPQSRPILIFCVSTYFIMMGILTLYTTYKEKGIFVVTIQKDPAGFVPDNVWEASSYLKKYDDKYNLVLTCKDGKTGTLREASLIKSVANFFDENGTLVYELLEPEISKLHNSLLADRKEK |
| Scp25 | ORTHOPTERA | *Acheta domesticus* | Locus_5447 | MASKNAKDKDNNQDKPVRINKWDGNAVKNALDDAVKEVLTTKFNYVEHFALMDGRLFMCGIAVGVAMFALLWDYLYPFPQSRPILIFCVSTYFIMMGILTLYTTYKEKGIFVVAIQKDPAGFVPDNVWEASSYLKKYDDKYNLVLSVKDGKSGACREATLVKSVANFIDENGTIVYELLEPEISKLHNTLLLSERKDK |
| Scp25 | ODONATA | *Ladona fulva* | Locus_2038 | MASKNSKESKEINNFEKPVKINKWDGSAVKNALDDAVKEVLTRRFNYQESFALMDGRLAMCGFAVTVAMFALVWDYLYPFPQSRPVLIFCVSTYFIMMGVLTLYTTYKEKGIFVVTLHKDPVGMSPDSVWEASSYLKKYDDKYNLALIYRDSKNGSVREATLTKSVACFFDENGVLLYDLLEPEVVKLHNSLLSEKKEK |
| Scp25 | ODONATA | *Libellula vibrans* | Locus_8212 | MASKSSKDSQAFQVYEKPVRINKWDGSAVKNALDDAVKEVLTRKFNYVESFALMDGRLAMCGFAVAVAMFALVWDYLYPFPQSRPVLIFCVSTYFIMMGVLTLYTTYKEKGIFVVTVQKDPAGFLPDSIWEASSYLKKYDDKYNLALLCRDGRTGAIREGTITKSVACFFDENGTLLYELLEPEVTKLHNGLLADKKDK |
| Spc25 | PHASMATODEA | *Sipyloidea sipylus* | Locus_6732_Transcript_4 | MSTKGGKDQDKPVKVNKWDGSAVKNALDDAVKEVLTKKFNYIESFALMDGRLAMCGIAVGVAMFALLWDYLYPFPQSRPILIFCVTAYFVMMGILTLYTTYKEKGIFVVAVQKDQAGFVPDNLWEASSYLKKFDDKYNLILSCKNGKTGARHEATLVKTVANFFDENGTLIYELLEPEVSKLHNSLLAERKEK |
| Scp25 | EPHEMEROPTERA | *Ephemera danica* | Locus_1602 | MSSKSVPADLKEENQPVKINKWDGSAIKNALDDGVKMALQRKFHYKENFALMDGRLAMCGVAVGVAMFAMLWDYLYPFPQSRPVLIFCVSCYFVMMGLLTLYTTYKEKGIFVVAVQQDPAGFNPDNIWEASSYMKKYDDKYNLVLSVKDGRTGLIKEASLTKSVACFFDTNGEMLYDALEAEIARVHNHLLADHKNK |

**Figure 2- supplemental source data 1**
